# Spectral Characteristics and Molecular Structure of (E)-1-(4-Chlorophenyl)-3-(4-(Dimethylamino)Phenyl)Prop-2-en-1-One (DAP)

**DOI:** 10.3390/ma14112766

**Published:** 2021-05-23

**Authors:** O. Aldaghri

**Affiliations:** Physics Department, College of Science, Imam Mohammad Ibn Saud Islamic University (IMSIU), Riyadh 13318, Saudi Arabia; odaghri@gmail.com

**Keywords:** laser dye DAP, ASE, superexciplex, molecular structure, DFT

## Abstract

In this work, a laser dye of (E)-1-(4-chlorophenyl)-3-(4-(dimethylamino)phenyl)prop-2-en-1-one (DAP) was synthesized and examined as a new laser medium. The compound DAP’s photophysical properties were investigated under the influence of solvents, concentrations, and pump power excitations. The absorption spectra showed a single band, and the shape of the spectra remained the same, regardless of the optical density. The fluorescence spectra showed a band around 538 nm; its intensity was inversely proportional to the concentration. DAP exhibits dual amplified spontaneous emission (ASE) bands at 545 and 565 nm under suitable pump power laser excitation and concentration. The results revealed that the ASE band at 565 nm is affected by solvents polarity, concentrations and pump power energies. This band could be attributed to the combination of two excited molecules and the solvent between them (superexciplex). Moreover, the molecular structure, the energy bandgap, and the total energy of DAP was calculated using density functional theory.

## 1. Introduction

Organic laser dye materials are commonly used in technology nowadays [1,2]. One of their applications is in electronic devices and optoelectronic sensors [3]. Conventional laser media are expensive, such as titanium-doped sapphire crystal [4]. On the contrary, laser dyes, such as rhodamine and coumarin series, are considered the cheapest laser medium materials, with output wavelength in the red and blue regions, respectively. Nevertheless, these dyes have low lasing efficiency and rapid photodegradation [5,6,7]. Researchers have been driven to manufacture many laser dyes to avoid these drawbacks. These synthesized materials are distinguished by their ease of synthesis, as well as their distinctive properties [8,9,10,11,12].

When a laser source optically excites certain organic molecules dissolved in organic solvents, these organic molecules will absorb the incoming photons and then emit either amplified spontaneous emission (ASE) or intensive light (laser) when the proper population inversion condition is satisfied [13]. The generated ASE is entirely dependent on the active medium, which is the organic molecule in this instance. Most dissolved organic molecules in organic solution have one fluorescence and ASE band, yet some dye molecules and conjugated polymers have dual ASE bands under certain conditions [14]. When an excited molecule combines with another molecule in the ground state, this process is called exciplex. Whereas two identical excited molecules combine with a solvent, the solvent acts as a bridge between two excited molecules, called a superexciplex [5,15,16].

Despite the great efforts that have been dedicated to improving the performance and efficiency of light-emitting organic lasing materials, the lack of information about photo-degradation and some organic dyes’ laser threshold remains a challenge for scientists [17,18,19,20,21]. Chalcone derivatives have a functionality of a carbonyl group in conjugation with a carbon-carbon double bond, which is known as α, β-unsaturated keto group, or enone function.

The dimethylamino-substituted chalcones have broad band spectra in the ultraviolet-visible (UV-Vis) range. This feature makes the chalcone a potential candidate for a tunable laser medium. In addition, these chalcones have shown high absorption coefficient results to achieve amplification light [18]. Chalcones show improved thermal, as well as photochemical stability, which makes them good dye laser media [22]. Nevertheless, such photo-physical characteristics are highly influenced by the attached functional groups (i.e., electron donating or withdrawing groups and the solvent environment). Chalcones with proper electron pulling functional groups on the two aryl rings had exhibited intrinsic fluorescence by affecting relevant parameters such as absorption and emission wavelengths, extinction coefficient and quantum yield [23]. The N,N dimethylamino group, attached to the para-position in ring B in chalcones, enhances its photophysical properties via intramolecular charge transfer. Among these chalcones, (E)-1-(4-chlorophenyl)-3-(4-(dimethylamino)phenyl)prop-2-en-1-one (DAP), has been chosen due to its unique optical and ASE properties. 

In this present study, investigations were carried out to explore the optical behavior of DAP in various organic solvents, concentrations, and pump power energies, in order to find the suitable conditions for the dual ASE peak to appear. When such an active medium is kept in a proper resonator, the laser band usually coincides with the fluorescence band. In contrast, DAP showed a new band that does not coincide with the steady-state fluorescence; this band might be due to combining two molecules in the excited state and the solvent play as a bridge. In addition, density functional theory (DFT) calculations are used to obtain the molecular structure, the energy bandgap, and the total energy of the material under study.

## 2. Materials and Methods

**Figure 1:**
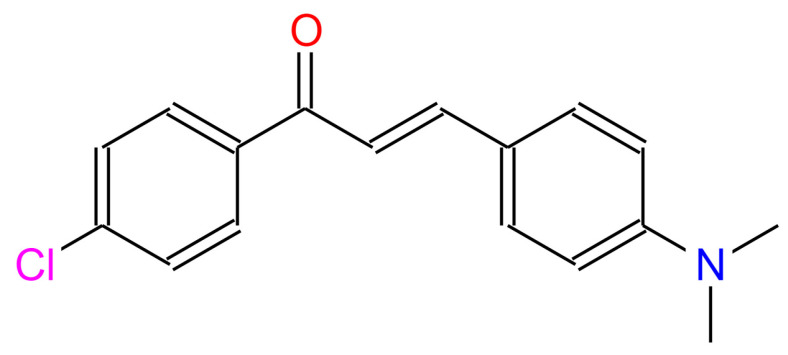
The molecular structure of DAP.

The DAP compound was synthesized using a reaction between 4-(dimethylamino)benzaldehyde and 4-chloroacetophenone in the presence of NaOH and alcohol as a laser dye material. Then, the compound was recrystallized from ethanol and washed with distilled water [24] (refer to the supplementary material for the product characterization). The molecular structure is displayed in Figure 1.

**Figure 2:**
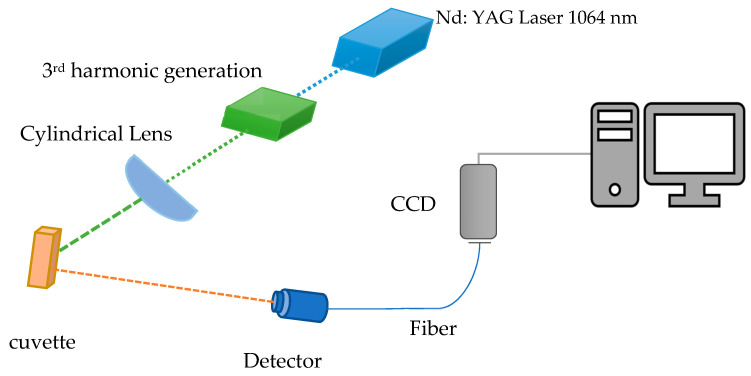
The experimental setup for the transverse excitation of DAP.

The DAP was dissolved in ten organic solvents (spectroscopic grade of 99.8% purity); Table 1 lists the used solvents. Under a wide range of concentrations, the optical properties, such as absorption and fluorescence, were recorded using a crystal quartz cuvette. The absorption spectra of DAP were measured for a wide range from 100–1000 nm using a Perkin-Elmer Lambda 950 UV-vis-NIR Spectrophotometer (Waltham, MA, USA), which has a double monochromator with holographic grating and double beam with ratio recording organized by a computer. For emission spectra, the Perkin-Elmer LS55 spectrofluorometer (Waltham, MA, USA) range was used in the range of 200 to 900 nm, at room temperature. The excitation wavelength was fixed at 355 nm for all samples. A quartz plano-cylindrical lens with a focal length of 5 cm is used to focus the UV laser, as a transverse pumping technique, on the sample [21]. The ASE beam is confined using optical fiber and analyzed by a charge-coupled device (CCD) camera, Solar M226 (Minsk, Belarus), as shown in Figure 2.

The theoretical calculation of DAP was carried out using DFT permed with Becke’s three parameter hybrid functional using the Lee–Yang–Parr correlation functional theory (B3LYP) [25,26,27] 6-311G** was utilized in the calculations as a basis set. All optimized calculations were carried out using Gaussian 09 quantum chemistry package (Wallingford, CT, USA) [28]. Both optimized structure draw and results visualization were carried out using GaussView 6.0 program (Wallingford, CT, USA) [28].

## 3. Results and Discussion 

### 3.1. The Steady-State of DAP

**Figure 3:**
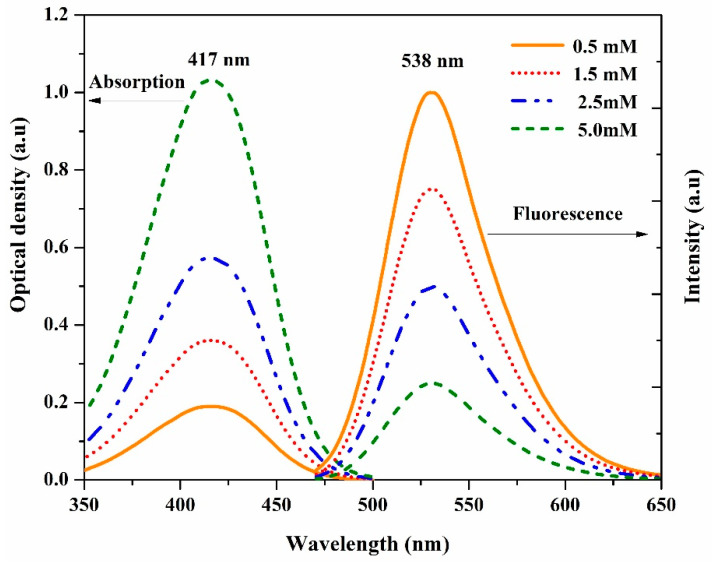
The absorption and fluorescence spectra of DAP in AN solvent for different concentrations.

The DAP was dissolved in acetonitrile (AN) under a wide range of concentrations (0.5 to 5 mM). The absorption spectra exhibited a single band at 417 nm, as shown in Figure 3. By increasing the concentration, no new peak appeared, which implies the absence of dimer formation in all used concentrations. The fluorescence spectra of DAP in AN showed a single peak at 538 nm, for all mentioned concentrations, as observed in Figure 3. One can observe that the intensity decreased with increasing the concentration. This behavior might be due to the association of two excited molecules in the excited state with the solvent (superexciplex stabilization in the high dielectric environment). This rare phenomenon was observed in few dyes and conjugated polymers [15,29].

The dissolved DAP in different solvents was prepared while the concentration for all solutions was fixed at 0.5 mM. The results show that the absorption and fluorescence peaks positions are affected by changing the solvent nature; for example, the absorption and the fluorescence of DAP in toluene were centered at 413 nm and 480 nm, respectively. In comparison, dimethylformamide, the absorption, and the fluorescence were located at 417 nm and 541 nm, respectively. The peak positions of the absorption and fluorescence of DAP in different organic solvents are recorded in Table 1. One can see that the solvent’s nature plays an essential function in the relative spectral peak position.

### 3.2. Stokes Shift

Stokes shift is the energy difference between the maximum peak of the absorption and the fluorescence corresponding to the same electronic transition. Herein, DAP in various organic solvents that have distinct dielectric constants was dissolved. While the concentration is fixed at 0.5 mM, there is a significant shift in the absorption and fluorescence spectra peak position. The Lippert–Mataga equation displays a linear dependence of the Stokes shift with the solvent polarizability (see Equations (1) and (2)) [30]:

(1) $$\Delta \nu \approx \frac{{\left(\mu_{e}-\mu_{g}\right)}^{2}\Delta_{f}}{a^{3}h\;c}+\mathrm{const}.,$$

(2) $$\mathrm{Dipole}\;\mathrm{factor}\;\Delta_{f}=\left[\frac{\left(\varepsilon -1\right)}{\left(2\varepsilon +1\right)}-\frac{\left([n^{2}-1\right)}{\left(2n^{2}+1\right)}\right],$$

where Δν is the difference between absorption and fluorescence peaks in wavenumber (cm^−1^). $\mu_{e}$ ($\mu_{g}$) referred to the dipole moment of the solute in the excited (ground) state. ε is the dielectric constant. n is the solvents refractive index, and ‘a’ is the Onsager cavity radius.

**Figure 4:**
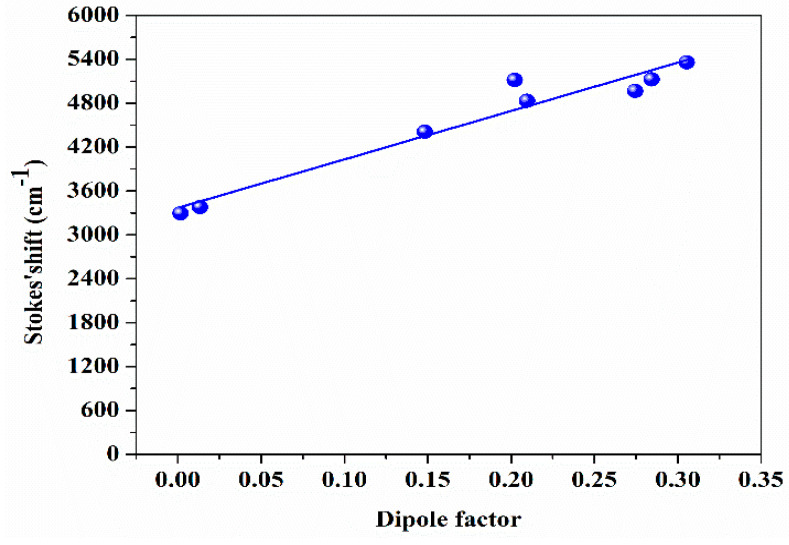
The Stokes shift of the DAP as a function of the dipole factor for different solvents.

This dipole factor in Equation (2) measures the dipole–dipole interaction between the solvents and the solute [13,31].The results show that DAP exhibited significant Stokes shifts (up to 5000 cm^−1^), as displayed in Figure 4. Upon comparing DAP with the rhodamine and coumarin series, it is found that the polarity of the DAP is greater by 150 orders of magnitude than the conventional organic dyes.

### 3.3. Quantum Yield of Fluorescence

One of the essential optical features is the fluorescence quantum yield ($\Phi_{f}$) of the luminescent molecule, which gives the number of emitted photons to the absorbed one. As the concentration was fixed at 0.5 mM, the quantum yields of used solutions were calculated using Equation (3) and recorded in Table 1.

(3) $$\Phi_{f}\left(s\right)=\Phi_{f}\left(r\right)\left(\frac{1-10^{-A_{r}}}{1-10^{-A_{S}}}\frac{n_{s}^{2}}{n_{r}^{2}}\right)\frac{\int I_{\;s}\left(\bar{\upsilon }\right)d\bar{\upsilon }}{\int I_{r}\left(\bar{\upsilon }\right)d\bar{\upsilon }}\;,$$

where the subscripts s (r) refer to sample (reference); the integrals over I represent the corrected fluorescence spectrum area; and A is the excitation wavelength absorbance.

### 3.4. Amplified Spontaneous Emission (ASE)

**Figure 5:**
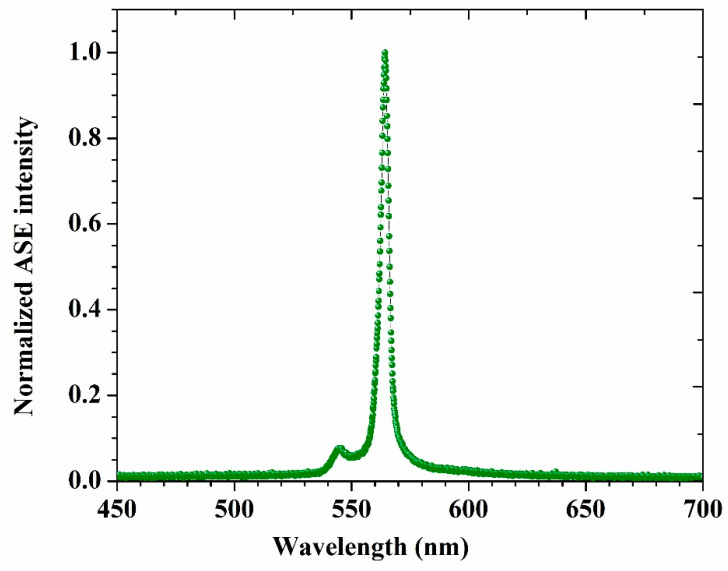
The ASE spectrum of the DAP in AN at 1.5 mM as fixed concentration.

A solution of 1.5 mM in AN, as a high polar solvent, was prepared and transversely excited using the third-harmonic generation of an Nd: YAG laser pulses (355 nm) with sufficient energy of 3 mJ; this is the lowest concentration and pumping power energy required to obtain the ASE spectrum. A dual ASE peak appeared, with two narrow spectral regions, one at 545 and another at 565 nm, with full width at half maximum (FWHM) of 8 nm for each peak (see Figure 5). The ASE peak at 545 nm corresponds to the fluorescence peak at 541 nm, and there is no fluorescence band at 565 nm corresponding to ASE at 565 nm. Based on the obtained results, one can attribute the peak at 565 nm to the presence of the superexciplex state [32,33,34,35].

**Figure 6:**
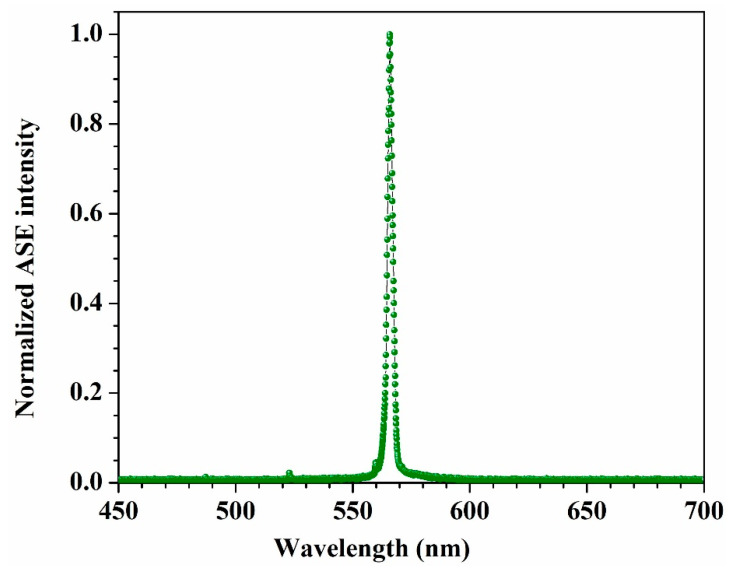
The ASE spectrum of the DAP in AN at 2 mM.

Only one ASE peak appearing at 565 nm was observed when the concentration increased to 2 mM (see Figure 6). This peak might be attributed to the superexciplex state. The disappearance of the 545 nm might be because all solute species were surrounded by the solvent’s species, due to the dipole–dipole induction process between the solvent and solute in the excited state.

No ASE peak was detected for non-polar solvents such as toluene, benzene, and acetic acid. In the case of the toluene and benzene, this may be due to their poor solubility, whereas acetic acid could be accounted for by the lone pair protonation in the N-dimethylamino group of DAP. For the solvents with hydrogen bonding, such as methanol or ethanol, the ASE was not observed. In this case, the hydrogen bonding in methanol deactivates the lone pair of the N-dimethylamino group. The other solvents, such as chloroform, maybe refer to the chlorine group in DAP, which prevents the presence of ASE.

In the case of other solvents, with intermediate polarity, such as acetone (tetrahydrofuran), the DAP shows one ASE peak at 547 (538) nm, which matches the fluorescence peak at 1.5 mM (see Table 1). The longer wavelength peak’s nonappearance means that the species at that wavelength do not form due to the solvent’s polarity.

**Figure 7:**
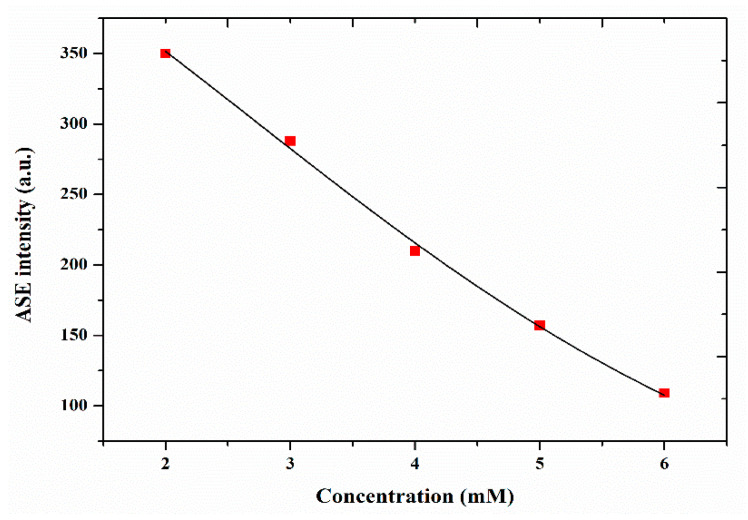
The dependence of the ASE peak intensity vs. the concentration of DAP in AN at 6 mJ.

Figure 7 shows the ASE intensities vs. concentrations of the DAP in AN, with pumping power excitation at 6 mJ. The results revealed that the ASE peak (565 nm) decreases rapidly as the concentration increases, which may be due to the association of two excited molecules in the excited state with the solvent (superexciplex). Miasojedovas et al. attributed the reduction in the ASE intensity vs. the increasing substituted perylene diimide derivatives PDI content to the reduced intermolecular separation that led to diminishing the optical losses and lowering of the ASE intensity [36].

**Figure 8:**
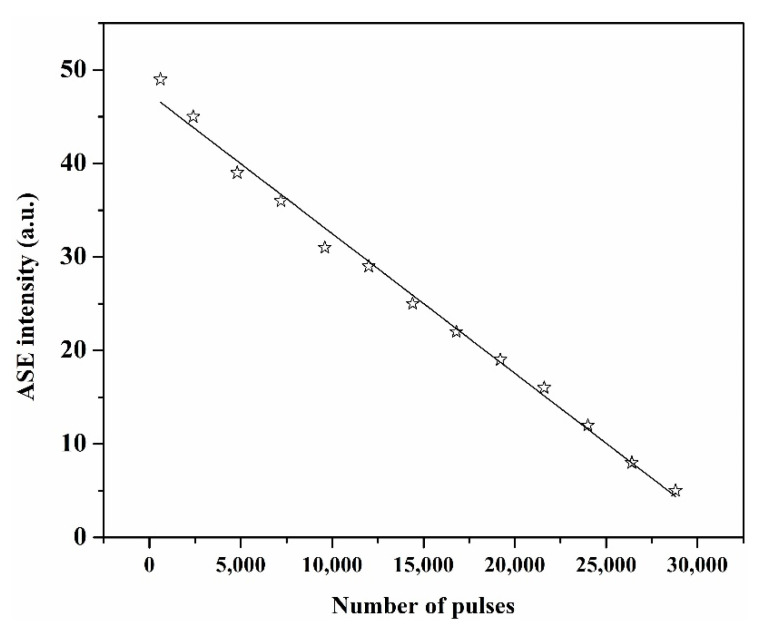
The emission stability of the 5 mM DAP in AN.

The ASE stability of the 5 mM solution of DAP in AN was measured under 6 mJ and 1 Hz pump power energy and repetition rate, respectively, using a 355 nm pulsed laser source. The solution was never circulated or stirred. After almost 4 h (15,000 pulses), the output intensity dropped 50% of its original performance (see Figure 8).

**Figure 9:**
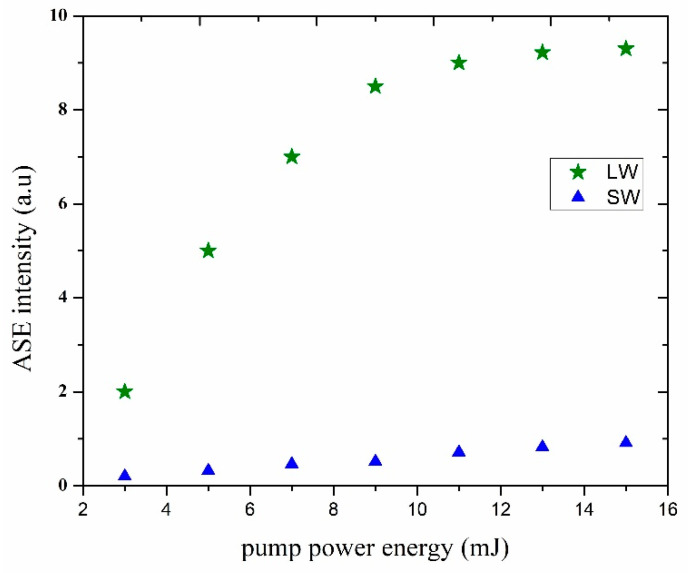
The ASE intensities of DAP in AN (at 1.5 mM as fixed concentration) as a function of pump power energy.

Figure 9 illustrates the increase of the ASE intensity as a function of the pumping power energy for a fixed 1.5 mM of DAP solution in AN. For a pump power energy from 3 to 15 mJ, the recorded intensities for the dual ASE peak were one for the short-wavelength (SW) at 545 nm, and another at 565 nm long-wavelength (LW). One can see the saturation of the LW intensity for pump power more than 9 mJ [5].

### 3.5. Theoretical Calculations

**Figure 10:**
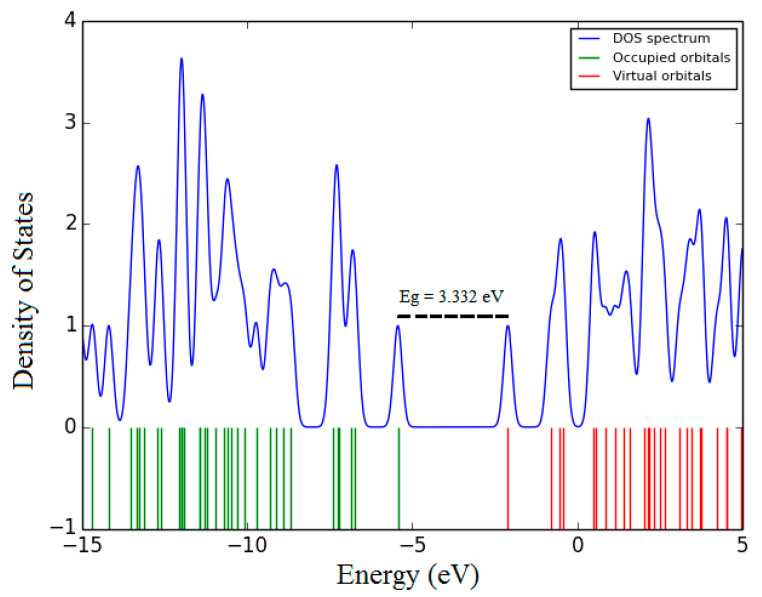
The density of states diagram of the DAP.

**Figure 11:**
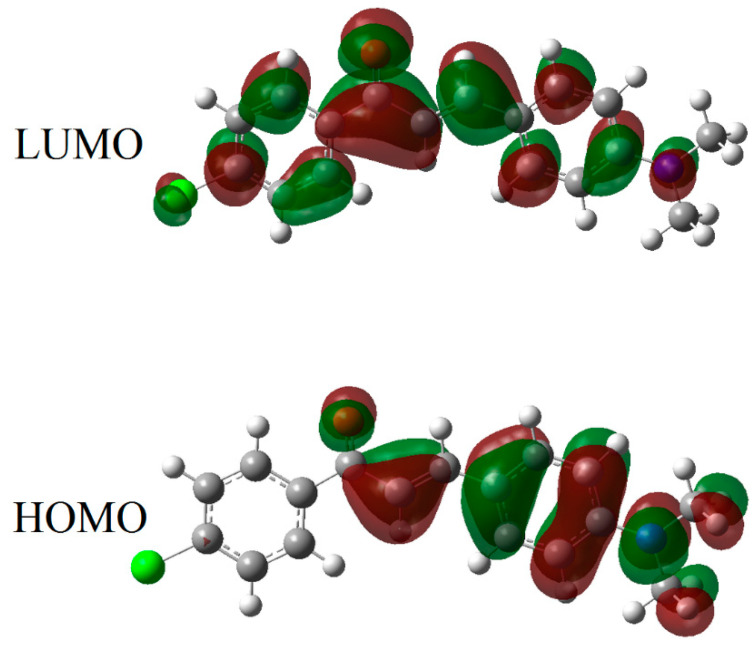
The molecular orbital (HOMO–LUMO) diagram of DAP.

The theoretical data, including the highest occupied molecule orbital (HOMO) and the lowest un-occupied molecular orbital (LUMO) transitions, molecule dipole moment (µ_D_), total energy (E_T_), and energy band gaps (E_g_), were calculated using DFT, as shown in Table 2. Figure 10 and Figure 11 show the density of states diagram and the HOMO–LUMO of DAP, respectively.

**Figure 12:**
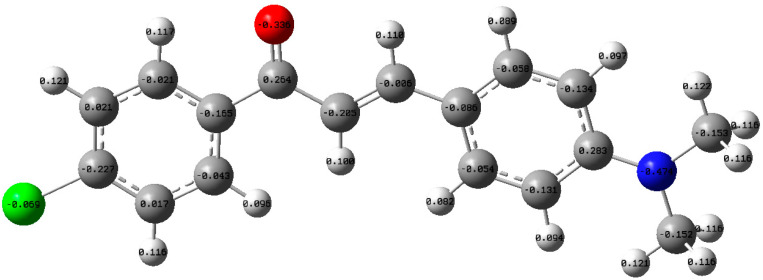
The Mulliken charges illustrated by the optimized structure of the DPA molecule.

Mulliken charge is used to illustrate the electron distribution between the atoms in specific molecules. The negative sign indicates that the atom has gained electrons, and the positive sign refers to electron loss. In addition, this method used to specify the type of bonds and dipole moment [37]. Herein, the electron distribution for DAP was performed using the Mulliken method, as shown in Figure 12.

## 4. Conclusions

After investigating the optical characteristics of the influence of solvent concentrations and solvent environments on DAP, the absorption spectra showed a single peak (located between 413 and 429 nm). The fluorescence showed one peak (between 480 and 542 nm) under the same operational conditions. The Stokes shift was calculated for these solvents (>3300 cm^−1^). The quantum yield varied with the nature of the solvent. Under the chosen pump power energy, the DAP, in a particular solvent and concentration, exhibit dual peaks around 545 (565) nm due to monomer (superexciplex). Superexciplex is favored in some solvents that have high polarity. For DAP, the DFT was used to compute the molecular orbitals, total energy, dipole moment, electron charges distribution, and the HOMO–LUMO transitions (3.332 eV energy band-gap).

## Figures and Tables

**Table 1:** The optical characteristics of DAP in different organic solvents.

Solvent	Dipole Factor	Absorption Peak (nm)	Fluorescence Peak (nm)	Short	Long	*Φ_f_* (%)
				ASE Peak (nm)		
Benzene	0.0016	420	487	-	-	16
Toluene	0.0132	413	480	-	-	12
Chloroform	0.1483	424	521	-	-	91
Acetic acid	0.2022	429	520	-	-	25
Tetrahydrofuran	0.2096	413	516	538	-	88
Acetone	0.2843	418	532	547	-	70
Ethanol	0.2887	425	535	-	-	40
Methanol	0.3086	427	533	-	-	20
Dimethylformamide	0.2744	427	541	546	566	95
Acetonitrile	0.3054	417	538	545	565	44

**Table 2:** The calculated chemical descriptors of the studied molecule.

E_T_ (eV)	E_HOMO_ (eV)	E_LUMO_ (eV)	E_g_ (eV)	µ_D_
−33,954.992	−5.432	−2.103	3.332	8.046

## Data Availability

Not applicable.

## Supplementary Materials

The Supplementary Materials are available online at https://www.mdpi.com/article/10.3390/ma14112766/s1, Figure S1: The XRD diffraction profile of DAP.
