# Supplementary material for: Spectral Characteristics and Molecular Structure of (E)-1-(4-Chlorophenyl)-3-(4-(Dimethylamino)Phenyl)Prop-2-en-1-One (DAP)
Source: Materials (Basel). 2021 May 23;14(11):2766. doi: 10.3390/ma14112766 (PMC8197161; doi:10.3390/ma14112766)
Supplement: Supplementary file 1 [file materials-14-02766-s001.zip › materials-1164296-supplementary.pdf]

*materials*

MDPI

*Supplementary Materials*

# Spectral characteristics and Molecular structure of (E)-1-(4-chlorophenyl)-3-(4-(dimethylamino)phenyl)prop-2-en-1-one (DAP)

**O. Aldaghri**

Physics Department, College of Science, Imam Mohammad Ibn Saud Islamic University (IMSIU), Riyadh 13318, Saudi Arabia; odaghri@gmail.com

The (E)-1-(4-chlorophenyl)-3-(4-(dimethylamino)phenyl)prop-2-en-1-one (DAP) compound was synthesized using a reaction between 4-(dimethylamino)benzaldehyde and 4-chloroacetophenone in the presence of NaOH and alcohol as a laser dye material. Then, the compound was recrystallized from ethanol and washed with distilled water [1,2]. The product was confirmed by FTIR, $^1$H NMR, and UV-Vis spectroscopy. The $V_{max}$: 1655 (C=O), 1583 cm$^{-1}$ (C=C); $^\delta$H (600 MHz, $CDCl_3$, $Me_4Si$) 3.03 (6H, s, $NMe_2$), 6.67 (2H, d, J= 9 Hz), 7.26 (1H, d, J=16 Hz), 7.43 (2H, d, J = 7 Hz), 7.53 (2H,d, J= 9 Hz), 7.77 (1H, d, J=16 Hz), 7.93 (2H, d, J= 7 Hz) ppm; $\lambda_{max}$ (Methanol 427 nm).

The crystallinity of DAP was investigated using XRD diffractometer (D8 Advance Bruker) using Cu–K$\alpha$ irradiation, $\lambda$ = 0.15406 nm, accelerating voltage is 40 kV, 15 mA and 20–80° as scanning angle. The estimation of the average crystallite size of the DAP was characterized utilizing an X-ray Diffractometer. The findings demonstrated that the peaks at various crystal planes of DAP manufactured agree exactly with the one prepared by the standard method, see Figure S1. The diffraction peaks are noted at 2 theta values equal to 10.63, and 25.64° corresponding to (010) and (100) planes with the 35.12 nm average crystallite size as obtained from Debye Scherer's equation [3,4].

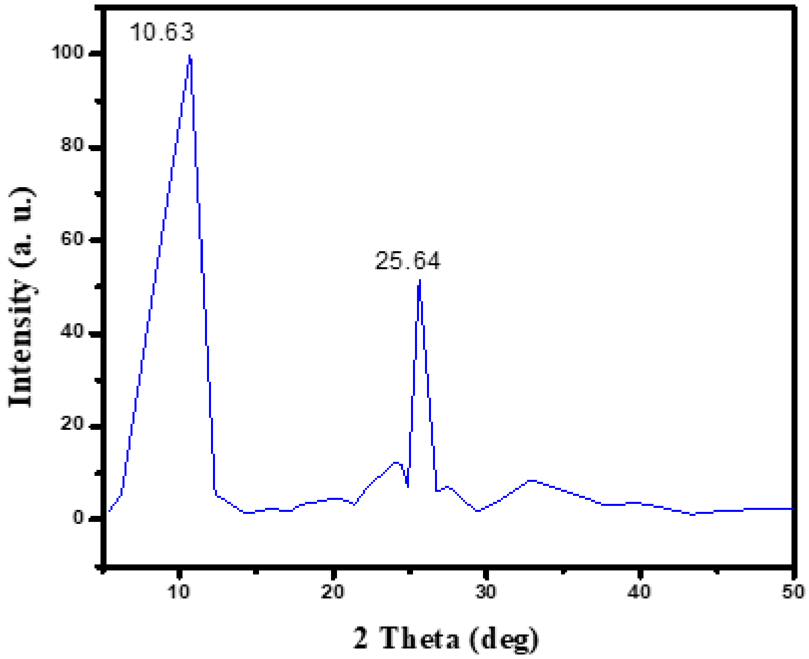


**Figure S1.** The XRD diffraction profile of DAP.

## References

1. Choudhary, N.A.; Kumar, A.; Juyal, V. Design, synthesis and evaluation of chalcone derivatives as anti-inflammatory, antioxidant and antiulcer agents. Letters in Drug Design & Discovery, 2012. 9(5): p. 479-488.
2. Elzupir, A.O., et al., Ultrasound irradiation promoted synthesis of chalcones, analogues, homologues and related furanyl containing compounds and their antibacterial activity. Int. J. Curr. Pharm. Res., 2013. 5(4): p. 23-25.
3. Modwi, A., et al., Effect of annealing on physicochemical and photocatalytic activity of Cu 5% loading on ZnO synthesized by sol–gel method. Journal of Materials Science: Materials in Electronics, 2016. 27(12): p. 12974-12984.
4. Jadhav, N.L., A.B. Pandit, and D.V. Pinjari, Green approach for the synthesis of chalcone (3-(4-fluorophenyl)-1-(4-methoxyphenyl) prop-2-en-1-one) using concentrated solar radiation. Solar Energy, 2017. 147: p. 232-239.
